# Ruxolitinib combined with azithromycin for scrub typhus-associated hemophagocytic lymphohistiocytosis in a child: a case report and narrative literature review

**DOI:** 10.3389/fped.2026.1852110

**Published:** 2026-06-15

**Authors:** Xingqiang Dong, Rongzhou Xiao, Chunyan Gao, Saihu Huang, Xiangying Meng, Xiangming Yan, Zhenjiang Bai, Shuiyan Wu

**Affiliations:** 1 Department of Critical Care Medicine, Childrenıs Hospital of Soochow University, Suzhou, China; 2 Pediatric Intensive Care Unit, Children’s Hospital of Soochow University, Suzhou, China; 3 Suzhou Key Laboratory of Pediatric Critical Care Medicine, Suzhou, China

**Keywords:** scrub typhus, hemophagocytic lymphohistiocytosis, ruxolitinib, azithromycin, child, JAK inhibitor

## Abstract

**Background:**

Scrub typhus-associated hemophagocytic lymphohistiocytosis (HLH) is a rare but life-threatening complication in children, with reported mortality of 11.9%–30%. Conventional immunomodulation with corticosteroids and intravenous immunoglobulin often provides insufficient control of the hyperinflammatory state, while etoposide-based chemotherapy carries significant toxicity. JAK1/2 inhibition targeting the interferon-gamma pathway represents a promising therapeutic strategy, but its application in scrub typhus-associated HLH has not been previously reported.

**Case presentation:**

A 5-year-11-month-old girl with no prior medical history presented with persistent fever, tachypnea, hepatosplenomegaly, and a 0.5 cm eschar in the left axilla after travel to Yunnan Province, China. Laboratory findings revealed pancytopenia (platelets 40× 10^9^/L), hyperferritinemia (>2,000 ng/mL), hypofibrinogenemia (1 g/L), and elevated interferon-gamma (135.48 pg/mL). Bone marrow aspiration demonstrated hemophagocytosis. Metagenomic next-generation sequencing confirmed Orientia tsutsugamushi infection. The patient met six of eight HLH-2004 diagnostic criteria. She was treated with oral ruxolitinib (5 mg twice daily) initiated on the day of admission, followed by intravenous azithromycin (10 mg/kg once daily) after confirmatory testing. Fever resolved within 72 h. Ruxolitinib was temporally associated with rapid clinical improvement, although causal attribution cannot be established due to concurrent therapies. By day 8, platelet count normalized to 240× 10^9^/L, ferritin declined to 1,246 ng/mL, and fibrinogen recovered to 2.4 g/L. The patient was discharged on day 13 with ruxolitinib tapered to 2.5 mg daily. At 3-month follow-up, she remained well with normal laboratory parameters.

**Literature review:**

Narrative literature review of 66 previously reported pediatric cases from Chinese and English databases (inception to May 2026) plus the present case revealed an overall mortality of 11.94% (8/67). Among these patients, 43 (64.2%) received corticosteroids, 34 (50.7%) received intravenous immunoglobulin, and only 3 (4.5%) received etoposide. The published cases suggest that absence or delay of anti-rickettsial therapy is associated with poor outcomes, though the evidence is limited by case-report bias and confounding.

**Conclusion:**

This is the first report of successful JAK1/2 inhibitor therapy in scrub typhus-associated HLH. This case raises a hypothesis worth investigating further—that ruxolitinib combined with azithromycin may achieve rapid disease control with good tolerability. Prospective studies are needed to evaluate the role of targeted JAK inhibition in infection-triggered HLH.

## Introduction

Hemophagocytic lymphohistiocytosis (HLH) is a life-threatening hyperinflammatory syndrome characterized by excessive activation of cytotoxic T lymphocytes and macrophages, leading to a cytokine storm and multiorgan dysfunction (1, 2). HLH can be classified as primary (genetic) or secondary (acquired), with infectious triggers representing a major cause of secondary HLH in children. Among infectious etiologies, scrub typhus—caused by the obligate intracellular bacterium Orientia tsutsugamushi—has emerged as an important trigger in endemic regions (3, 4). Scrub typhus is endemic in the Asia-Pacific region, including China, India, and Southeast Asian countries, with an estimated one million cases occurring annually. However, increased international travel has led to its recognition as an emerging global health concern. Scrub typhus typically presents with fever, eschar, lymphadenopathy, and hepatosplenomegaly, but its nonspecific manifestations often delay diagnosis, particularly in non-endemic areas (5). Severe scrub typhus can be associated with multiorgan dysfunction, acute respiratory distress syndrome, and mortality rates as high as 30% if untreated (5).

The incidence of HLH among pediatric scrub typhus patients is not negligible. Basu and colleagues (6) reported that 18 of 58 (31%) children with scrub typhus developed HLH. The mortality of scrub typhus-associated HLH ranges from 6.7% to 30% in published series (7, 8), with delayed anti-rickettsial therapy being the strongest predictor of poor outcome.

Current management of scrub typhus-associated HLH rests on two pillars: effective anti-rickettsial antibiotics and immunomodulation. Doxycycline is the first-line antibiotic, and current CDC guidance recommends doxycycline for suspected scrub typhus in all ages, including children under 8 years, noting that short courses do not cause dental staining (9). Macrolides such as azithromycin serve as alternative agents. For immunomodulation, corticosteroids and intravenous immunoglobulin (IVIG) are widely used (7, 10, 11). However, severe cases may require etoposide-based chemotherapy (HLH-94/2004 protocol) (12), which carries substantial risks of myelosuppression, secondary infections, and long-term complications in children.

Recent advances have identified interferon-gamma (IFN-γ) as a key driver of the cytokine storm, with signal transduction occurring via the JAK-STAT pathway (13, 14). Ruxolitinib, a selective JAK1/2 inhibitor, has demonstrated efficacy in murine HLH models (15) and in children with EBV-associated and primary HLH (16, 17). However, its application in scrub typhus-associated HLH has not been previously reported.

Here we present the first case of ruxolitinib treatment in pediatric scrub typhus-associated HLH, accompanied by a narrative literature review of 67 cases to contextualize this novel therapeutic approach.

## Methods

### Literature search and review approach

We conducted a narrative literature review (rather than a formal systematic review) to summarize published pediatric cases. We searched PubMed, China National Knowledge Infrastructure (CNKI), and Wanfang databases for articles published from database inception to May 2026. The search strategy combined the following terms: (“scrub typhus” OR “Orientia tsutsugamushi” OR “tsutsugamushi”) AND (“hemophagocytic lymphohistiocytosis” OR “HLH” OR “hemophagocytic syndrome”) AND (“child” OR “pediatric” OR “children”). No language restrictions were applied.

### Inclusion and exclusion criteria

Studies were included if they: (1) reported pediatric patients (aged 0–18 years) with confirmed scrub typhus infection; (2) met the HLH-2004 diagnostic criteria; and (3) provided sufficient clinical and treatment data. Case reports, case series, and observational studies were eligible. We excluded adult studies, animal studies, reviews without original case data, and duplicate publications.

### Data extraction

Two authors independently extracted the following data: patient demographics, clinical presentation, diagnostic methods, antibiotic therapy, immunomodulatory therapy (corticosteroids, IVIG, etoposide, ruxolitinib, or others), laboratory parameters, treatment outcomes, and follow-up status. Discrepancies were resolved by consensus.

### Case presentation

We additionally report a single case of scrub typhus-associated HLH treated at Children's Hospital of Soochow University (Suzhou, China) in July 2025. The patient's legal guardian provided written informed consent for publication of clinical data and images. The study was conducted in accordance with the Declaration of Helsinki and approved by the Institutional Review Board of Children's Hospital of Soochow University (approval No:2024cs160).

## Results

### Case presentation

A previously healthy 5-year-11-month-old girl presented with a 10-day history of fever (peak 39.6 °C) that began during travel to Yunnan Province, China. She had received empirical cephalosporin therapy for 2 days without improvement. Two days before admission, she developed tachypnea and lethargy.

**Figure 1:**
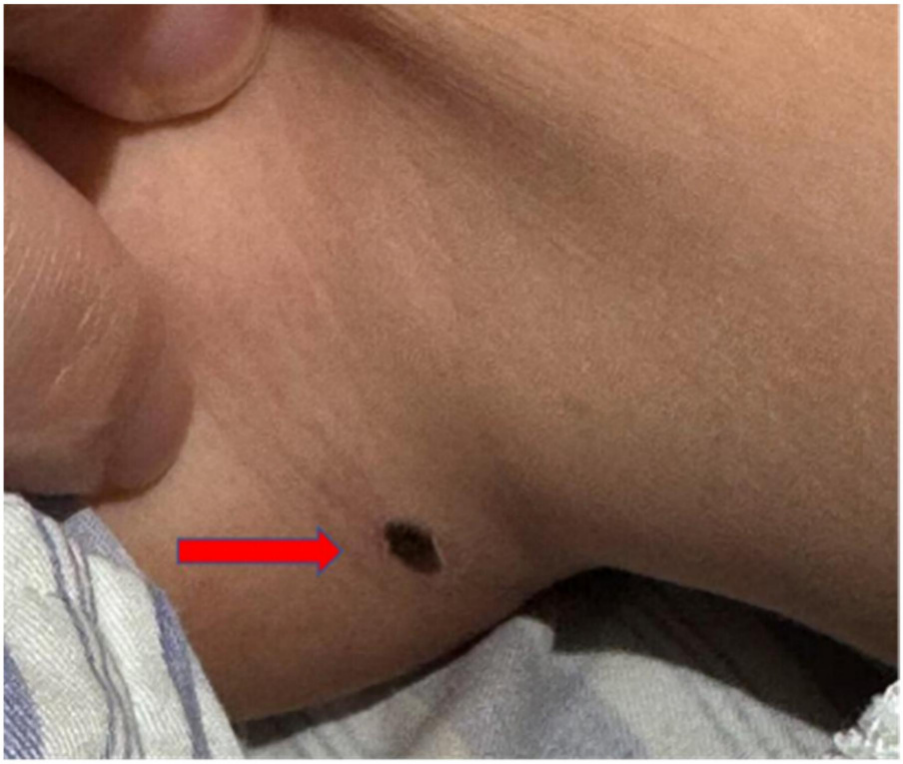
Clinical photograph showing the eschar in the left axilla (0.5 cm diameter).

On physical examination at admission, the patient had a temperature of 37.9 °C, heart rate 106/min, respiratory rate 34/min, blood pressure 100/73 mmHg, and oxygen saturation 94% on 2 L/min nasal cannula. She appeared pale and lethargic. Hepatomegaly (3 cm below the right costal margin) and splenomegaly (2 cm below the left costal margin) were noted. A 0.5 cm eschar was observed in the left axilla (Figure 1). Scattered, old skin rashes and hand-foot swelling were also present.

**Figure 2:**
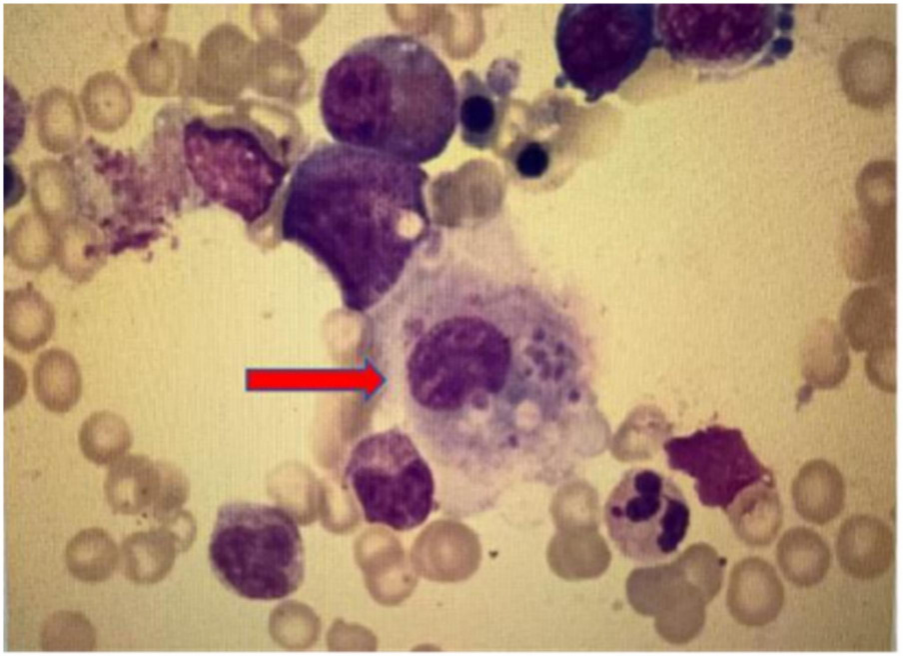
(Wright–Giemsa stain, ×400) bone marrow aspirate demonstrating hemophagocytosis (arrow indicates a hemophagocytic macrophage engulfing erythrocytes and platelets).

Laboratory findings revealed pancytopenia (hemoglobin 97 g/L, platelets 40 × 10^9^/L), hyperferritinemia (>2,000 ng/mL), hypofibrinogenemia (1 g/L), transaminitis (aspartate aminotransferase 142.7 U/L, alanine aminotransferase 70.4 U/L), and hypoalbuminemia (24.1 g/L). Serum cytokine profiling showed markedly elevated IFN-γ (135.48 pg/mL). Bone marrow aspiration revealed hemophagocytosis (Figure 2). Metagenomic next-generation sequencing (mNGS) of blood confirmed O. tsutsugamushi infection. mNGS details: whole blood sample collected on 07-27-2025; result returned on 07-29-2025 (∼48 h); 16 specific reads for O. tsutsugamushi; no other significant pathogens detected. The patient met six of eight HLH-2004 diagnostic criteria (see Table 1 for full documentation).

**Table 1:** HLH-2004 diagnostic criteria fulfilled by the patient.

Criterion	Patient value	Fulfilled
Fever	Persistent >7 days, peak 39.6 °C	Yes
Splenomegaly	Palpable 2 cm below costal margin	Yes
Cytopenias (≥2 lineages)	Platelets 40 × 10^9^/L, Hb 97 g/L (ANC 1.57 × 10^9^/L—not below 1.0)	Yes (platelets + Hb)
Hyperferritinemia	>2,000 ng/mL	Yes
Hypofibrinogenemia	1.0 g/L	Yes
Hemophagocytosis in bone marrow	Present (Figure 2)	Yes
Low/absent NK cell activity	Not tested	No
Elevated sCD25 (IL-2 receptor)	Not tested	No

Total fulfilled: 6/8.

#### Diagnostic assessment and differential diagnosis

We considered and excluded the following: primary/familial HLH (no family history or known mutations), EBV-HLH (EBV DNA positive in whole blood but negative in plasma, indicating past infection rather than active disease), Kawasaki disease/MIS-C (no typical mucocutaneous changes, normal coronary arteries), macrophage activation syndrome (no underlying rheumatologic disease, ANA 1:100 non-specific), and hematologic malignancy (no blasts on bone marrow aspirate).

#### Therapeutic intervention

Treatment was initiated immediately upon admission. The patient received high-flow nasal cannula oxygen (20 L/min, FiO_2_ 50%), intravenous piperacillin-tazobactam (day 1–3), and oral ruxolitinib (5 mg twice daily) starting on day 1. Ruxolitinib dose: 5 mg bid (weight 17.5 kg, BSA ∼0.7 m^2^, ∼0.3 mg/kg/dose). This dose was based on pediatric HLH studies by Wei et al. (15) and Zhang et al. (16). Use was off-label; written informed consent was obtained from the legal guardian. Intravenous azithromycin (10 mg/kg once daily) was added on day 3 after confirmatory mNGS results and continued until day 13 (11 days). She then received oral azithromycin (0.1 g daily) from day 14 to day 20 (7 days), for a total of 18 days of therapy. The delay in anti-rickettsial therapy was due to initial diagnostic uncertainty and the time required for mNGS results. Empirical doxycycline was not given because of parental concerns and local practice favoring azithromycin in young children, consistent with Chinese expert consensus (11). Supportive care included hepatoprotective agents and albumin infusion.

#### Safety monitoring

Daily complete blood counts, liver and renal function tests, coagulation profile, and infection markers were performed. No adverse events (myelosuppression, bleeding, secondary infection, or viral reactivation) were observed.

Fever resolved completely on day 3 of ruxolitinib therapy. Respiratory status improved progressively, allowing de-escalation to low-flow oxygen by day 4 and transfer to the general ward on day 5. Laboratory parameters showed substantial improvement: platelet count increased from 40 × 10^9^/L to 240× 10^9^/L by day 8, ferritin declined from >2,000 to 1,246 ng/mL by day 8 and normalized by day 13, and fibrinogen recovered from 1 to 2.4 g/L by day 8 (Table 2). Follow-up data are shown in Table 3. Liver function tests and albumin levels also improved. The patient was discharged on day 13 in good clinical condition, with ruxolitinib tapered to 2.5 mg daily. Ruxolitinib was discontinued at 4-week follow-up after all hematologic parameters remained normal.

**Table 2:** Longitudinal changes in key HLH-related laboratory parameters.

Parameter (unit)	Day 1	Day 3	Day 5	Day 8	Day 13	Reference range
Platelets (×10^9^/L)	40	59	110	240	310	150–450
Ferritin (ng/mL)	>2,000	1,890	1,520	1,246	105	15–150
Fibrinogen (g/L)	1.0	1.6	2.1	2.4	2.8	1.8–4.0
ALT (U/L)	70.4	55.2	42.1	32.9	21.0	10–40
AST (U/L)	142.7	98.3	65.4	38.8	35.0	10–40
Albumin (g/L)	24.1	28.2	32.5	41.2	48.8	35–50
Triglycerides (mmol/L)	1.85	–	–	1.92	2.13	<1.7

**Table 3:** Follow-up data at discharge, 4 weeks, and 3 months.

Parameter	Discharge (Day 13)	4 weeks	3 months	Reference
Hemoglobin (g/L)	120	125	128	110–140
Platelets (×10^9^/L)	310	402	380	150–450
WBC (×10^9^/L)	6.85	7.20	6.90	5–12
Ferritin (ng/mL)	105	68	52	15–150
Fibrinogen (g/L)	2.8	3.1	3.0	1.8–4.0
ALT (U/L)	21.0	18.5	16.0	10–40
CRP (mg/L)	<0.5	<0.5	<0.5	<5

No adverse events, no relapse, and no recurrent fever or infections were reported.

#### Patient perspective

The child's mother reported that the family initially did not appreciate the severity of the illness. After admission and explanation of the potential benefits and risks of off-label ruxolitinib, the guardian provided written consent. The mother noted that the child's fever subsided and mental state improved markedly within 3 days, which greatly relieved the family's anxiety. At the 3-month follow-up, the family expressed satisfaction with the treatment outcome and absence of any lasting problems.

### Literature review results

Our search identified 66 previously reported pediatric cases of scrub typhus-associated HLH from 10 eligible studies (6–8, 10, 17–22). Combined with the present case, a total of 67 patients were analyzed. All cases originated from Asia, including China (45 cases), India (20 cases), and South Korea (2 cases). Patient ages ranged from 2 months to 12 years.

Across the 67 cases, the most frequently used antibiotic was doxycycline (40 cases, 59.7%), followed by macrolides (13 cases, 19.4%) and chloramphenicol (11 cases, 16.4%). Among the 5 patients who did not receive any anti-rickettsial therapy (due to misdiagnosis), 4 died, yielding a mortality rate of 80% in untreated patients compared with 8. 1% among those who received appropriate antibiotics. This difference underscores the critical importance of timely pathogen-directed therapy.

For immunomodulation, corticosteroids were administered to 43 patients (64.2%) and IVIG to 34 patients (50.7%). Only 3 critically ill patients (4.5%) received etoposide-based chemotherapy. The overall mortality was 11.94% (8/67). The published cases suggest that absence or delay of anti-rickettsial therapy is associated with poor outcomes, though the evidence is limited by case-report bias and confounding (6–8). Demographic characteristics, treatment, outcomes, and hospital stay of the 67 patients are summarized in Table 4. A detailed clinical timeline is presented in Table 5.

**Table 4:** Demographic characteristics, treatment, outcomes, and length of hospital stay of 67 pediatric patients with scrub typhus-associated HLH.

Author (year)	Country	*n*	Sex (M:F)	Age	Antibiotic	Immunomodulation	Outcome	Hospital stay (days)
Jayakrishnan et al. (2011) (17)	India	1	1:0	5 years	Doxycycline	None	Survived	NR
Han et al. (2012) (18)	Korea	1	1:0	9 years	Doxycycline	IVIG	Survived	NR
Kwon et al. (2013) (19)	Korea	1	1:0	8 months	Clindamycin → chloramphenicol	IVIG, steroids	Survived	NR
Kong et al. (2014) (7)	China	19	9:10	11 mo–10 y	Doxycycline (15), unknown (4)	IVIG (8), steroids (14)	4 deaths	NR
Pazhaniyandi et al. (2015) (20)	India	1	1:0	2 months	Doxycycline	IVIG, steroids	Survived	NR
Zhou et al. (2016) (21)	China	3	1:2	2–5 years	Chloramphenicol	Steroids (3)	Survived	NR
Jin et al. (2016) (22)	China	6	6:0	8 mo–11 y	Azithromycin (2), doxycycline (4)	IVIG (5), steroids (6)	1 death	NR
Ke (2018) (10)	China	1	1:0	9 months	Azithromycin	IVIG, steroids	Survived	NR
Basu et al. (2021) (6)	India	18	ND	ND	Doxycycline (18)	IVIG (4), steroids (6)	1 death	NR
Lu et al. (2023) (8)	China	15	9:6	5.01 ± 3.82 y	Doxycycline (15)	IVIG (13), steroids (15)	2 deaths	NR
Present case (2025)	China	1	0:1	5 y 11 mo	Azithromycin	Ruxolitinib	Survived	**13**

ND, not described; IVIG, intravenous immunoglobulin; y, years; mo, months; NR, not reported.

Bold values indicate patients who died.

**Table 5:** Clinical timeline of the present case (CARE-compliant).

Time point	Illness day	Hospital day	Key event
2025-07-16	Day 1	_—_	Fever onset, peak 39.6 °C; headache, abdominal pain
2025-07-18	Day 3	_—_	First medical visit (Suoshan Town Hospital); empiric cephalosporin started
2025-07-20	Day 5	_—_	Return to Zhangjiagang from Yunnan; fever persisted
2025-07-23	Day 8	_—_	Zhangjiagang Aoyang Hospital visit; platelet 60 × 10^9^/L; chest x-ray showed bronchopneumonia
2025-07-25	Day 10	_—_	Admitted to Zhangjiagang People's Hospital; platelet 40 × 10^9^/L; ferritin >2,000ng/mL; fibrinogen 1 g/L
2025-07-26	Day 11	Day 1	Transferred to our hospital; eschar identified; bone marrow aspiration performed; ruxolitinib 5 mg bid started; piperacillin-tazobactam started; HFNC oxygen (20 L/min, FiO_2_ 50%)
2025-07-27	Day 12	Day 2	mNGS blood sample collected; respiratory distress noted
2025-07-28	Day 13	Day 3	Fever resolved;intravenous azithromycin (10 mg/kg/day) started (continued for 11 days); oxygen requirement decreased
2025-07-29	Day 14	Day 4	mNGS result returned (O. tsutsugamushi, 16 reads; EBV, 9 reads); transferred to general ward
2025-07-30	Day 15	Day 5	Ruxolitinib reduced to 5 mg daily; off oxygen
2025-08-01	Day 17	Day 7	Intravenous azithromycin continued; ruxolitinib 5 mg daily
2025-08-03	Day 19	Day 9	Ruxolitinib reduced to 2.5 mg daily
2025-08-07	Day 23	Day 13	Azithromycin switched to oral (0.1 g daily, continued for 7 more days); discharged; ruxolitinib 2.5 mg daily continued
2025-09-04	Day 51	Week 4	Follow-up visit: ruxolitinib discontinued; labs normal
2025-11-04	Day 111	Month 3	Follow-up visit: clinically well; all laboratory parameters normal

Total duration of azithromycin therapy: 18 days (11 days IV + 7 days oral).

## Discussion

This case-based review raises a hypothesis regarding the use of ruxolitinib, a JAK1/2 inhibitor, in pediatric scrub typhus-associated HLH. The patient demonstrated rapid clinical improvement, with fever resolution within 72 h and normalization of HLH biomarkers by day 8, suggesting that targeted JAK inhibition effectively controls the hyperinflammatory state while allowing anti-rickettsial therapy to clear the pathogen.

### Mechanistic rationale for JAK inhibition in HLH

The pathogenesis of HLH centers on a dysregulated immune response driven by excessive activation of CD8⁺ T lymphocytes and macrophages, leading to a cytokine storm. IFN-γ has been identified as a central pathogenic cytokine (1, 2). IFN-γ exerts its effects primarily through the JAK-STAT signaling pathway. Upon binding to its receptor, IFN-γ activates JAK1 and JAK2, which phosphorylate STAT1, leading to nuclear translocation and transcription of proinflammatory genes. Ruxolitinib is a potent and selective inhibitor of JAK1/2 that blocks this signaling cascade at its source (15–17).

In our patient, serum IFN-γ levels were markedly elevated at presentation (135.48 pg/mL), providing a strong mechanistic rationale for targeted JAK inhibition. The rapid clinical and laboratory response observed after ruxolitinib initiation directly supports the hypothesis that blocking the JAK-STAT pathway effectively controls the hyperinflammatory state in scrub typhus-associated HLH.

### Rationale for choosing ruxolitinib and comparison with conventional immunomodulatory approaches

We selected ruxolitinib over conventional first-line therapies (corticosteroids and IVIG) for several reasons: (1) the patient presented with severe hyperinflammation (ferritin >2,000 ng/mL, hypofibrinogenemia, cytopenias) and was at high risk for progression to multiorgan failure; (2) although corticosteroids are widely used, they provide non-specific immune suppression and may be insufficient to control severe cytokine storms; (3) etoposide, while effective in HLH, carries substantial risks of myelosuppression, secondary infections, and potential long-term complications in young children; and (4) recent pediatric studies have shown acceptable safety and efficacy of ruxolitinib in EBV- HLH and primary HLH (15, 16).

Current guidelines for secondary HLH recommend corticosteroids as first-line immunomodulatory therapy, often in combination with IVIG (2). However, these agents provide non-specific immune suppression and may be insufficient to control severe cytokine storms. In our literature review, although 64.2% of patients received corticosteroids and 50.7% received IVIG, the overall mortality remained 11.94% (6–8, 22), and 4.5% of patients required escalation to etoposide chemotherapy (12).

Etoposide, a topoisomerase II inhibitor, is the backbone of HLH-94/2004 protocols and has demonstrated efficacy in HLH (12). However, its use is associated with significant toxicities, including dose-limiting myelosuppression, increased risk of secondary infections, and potential for secondary malignancies. In infection-triggered HLH, particularly in young children, the risk-benefit ratio of etoposide is particularly concerning. The HLH-94 study reported treatment-related mortality of approximately 8% in children receiving etoposide-based regimens (12). Moreover, etoposide may compromise the host's ability to clear the underlying infection, as it targets rapidly dividing cells including activated lymphocytes involved in pathogen defense.

Ruxolitinib offers several theoretical advantages. Its oral formulation simplifies administration, and at therapeutic doses it does not typically cause significant myelosuppression; in fact, our patient's platelet count improved during treatment, consistent with its known effects on inflammatory thrombocytopenia (15, 16). However, a legitimate concern is whether JAK inhibition might impair host defense against the obligate intracellular bacterium O. tsutsugamushi. In our patient, mNGS showed no evidence of persistent or disseminated infection, and no relapse occurred during follow-up, suggesting that short-course ruxolitinib combined with effective anti-rickettsial therapy is safe in this context. This observation remains preliminary and requires further study.

### Antibiotic strategy considerations

In the present case, azithromycin was not started until day 3 after mNGS confirmation. The delay was due to initial diagnostic uncertainty (the eschar was noted on admission but travel history to an endemic area was obtained gradually) and the time required for mNGS results. The patient had previously received empirical cephalosporin without improvement, and other bacterial pneumonia was initially considered. Although doxycycline is the first-line antibiotic for scrub typhus and current CDC guidance recommends its use in all ages, including children under 8 years (short courses do not cause dental staining) (9), we chose azithromycin because the patient's guardian expressed concerns about doxycycline and because local Chinese expert consensus (11) supports macrolides as an alternative in young children. Ideally, empirical anti-rickettsial therapy should be initiated promptly when scrub typhus is suspected; this case illustrates the importance of early recognition.

### Comparison with previously reported JAK inhibitor use in HLH

Ruxolitinib has been studied in other forms of HLH. Zhang and colleagues (16) conducted a prospective study of ruxolitinib response-based stratified treatment for pediatric HLH, demonstrating that ruxolitinib monotherapy achieved remission in a substantial proportion of patients with EBV-associated and primary HLH, allowing many to avoid etoposide. Wei and colleagues (15) reported successful treatment of 5 children with refractory EBV-HLH and 2 children with primary HLH using ruxolitinib, with manageable toxicity. Our case extends these findings to rickettsial infection-triggered HLH, broadening the potential indications for JAK inhibitor therapy in secondary HLH.

### Clinical implications and recommendations

Our findings have several important clinical implications. First, they reinforce the critical importance of timely anti-rickettsial therapy in scrub typhus-associated HLH. Among the 5 patients in our literature review who did not receive appropriate antibiotics, 4 died (80% mortality), compared with 8. 1% mortality among those who received antibiotics (6–8). The published cases suggest that absence or delay of anti-rickettsial therapy is associated with poor outcomes, though the evidence is limited by case- report bias and confounding.

Second, our case suggests that ruxolitinib may be a valuable addition to the immunomodulatory armamentarium for infection-triggered HLH. For patients with severe disease who do not respond adequately to corticosteroids or IVIG, ruxolitinib offers a targeted alternative to etoposide. Given its favorable safety profile (15, 16), ruxolitinib might also be considered earlier in the treatment algorithm, potentially preventing progression to etoposide-requiring severe disease.

Third, the availability of mNGS for rapid pathogen identification facilitated timely diagnosis in our case. For patients presenting with fever and suspected HLH of unknown etiology, mNGS can rapidly identify the underlying infectious trigger, enabling targeted anti-infective therapy and appropriate immunomodulation.

### Limitations

This study has several limitations. First, it is based on a single case report with a literature review rather than a prospective controlled trial. Causal attribution of the favorable outcome to ruxolitinib cannot be definitively established, as the patient also received azithromycin and supportive care. The temporal association is suggestive but not proof of efficacy.

Second, the literature review is narrative and included only published cases, introducing potential publication bias. Cases with favorable outcomes are more likely to be reported, potentially overestimating treatment success rates. Moreover, we did not perform a formal quality assessment of included studies.

Third, long-term safety data on ruxolitinib use in young children are limited. Although our patient tolerated the medication well and had normal laboratory parameters at 3-month follow-up, longer follow-up in larger cohorts is needed to fully characterize the safety profile. Additionally, we did not measure soluble IL-2 receptor (sCD25) or NK cell activity, which are part of the HLH- 2004 criteria; this limits complete diagnostic documentation.

Fourth, the optimal dosing and duration of ruxolitinib for pediatric HLH remain to be determined. We used a starting dose of 5 mg twice daily based on published pediatric studies, tapering to 2.5 mg daily at discharge and discontinuing at 4 weeks. Further studies are needed to establish evidence-based dosing guidelines.

### Future directions

Prospective studies are needed to evaluate the efficacy and safety of ruxolitinib as first-line or early second-line immunomodulation for infection-triggered HLH. Biomarker-driven approaches, using IFN-γ levels or other cytokines to identify patients most likely to benefit from JAK inhibition, could enable personalized treatment strategies. Additionally, comparative effectiveness studies of ruxolitinib vs. conventional therapies would help define its optimal role in the treatment algorithm.

## Conclusion

This case raises a hypothesis that ruxolitinib combined with azithromycin may be a safe and effective immunomodulatory strategy for pediatric scrub typhus-associated HLH. The treatment was temporally associated with rapid disease control and excellent tolerability, offering a targeted, chemotherapy-sparing option that warrants further investigation in prospective studies.

## Data Availability

The datasets presented in this study can be found in online repositories. The names of the repository/repositories and accession number(s) can be found in the article/Supplementary Material.
